# Evaluation of the survival of homofermentative *Lacticaseibacillus casei* subsp. *casei* in fermented milk matrix enriched with non-digestible natural fibers

**DOI:** 10.1007/s13197-023-05698-z

**Published:** 2023-03-29

**Authors:** Emmanuel Iván Morales-Ríos, Hulme Ríos-Guerra, Judith Espinosa-Raya, Raquel Gómez-Pliego

**Affiliations:** 1 Laboratorio de Neurofarmacología, Escuela Superior de Medicina, Instituto Politécnico Nacional, Plan de San Luis y Díaz Mirón S/N, Miguel Hidalgo, C.P. 11340 Ciudad de México, México; 2 Departamento de Ciencias Químicas, Sección de Química Orgánica, Facultad de Estudios Superiores Cuautitlán, Campo 1, Universidad Nacional Autónoma de México, Av. 1 de Mayo S/N, Santa María de Guadalupe las Torres, Cuautitlán Izcalli, C.P. 54740 Estado de México, México; 3 Departamento de Ciencias Químico-Biológicas, Sección de Ciencias de La Salud Humana, Facultad de Estudios Superiores Cuautitlán, Campo 1, Universidad Nacional Autónoma de México, Av. 1 de Mayo S/N, Santa María de Guadalupe las Torres, Cuautitlán Izcalli, C.P. 54740 Estado de México, México

**Keywords:** Probiotics microorganism, Non-digestible prebiotic fibers, *L. casei* bacteria, Fermented milk, Physicochemical parameters

## Abstract

The physicochemical changes induced by seven different dietary fibers (oat, bamboo, pea, inulin, apple, potato, and wheat) during storage and their effects on the survival of homofermentative *Lacticaseibacillus casei* subsp.* casei* (*L. casei*) in fermented milk matrix were analyzed. For this, an experimental study of the effect of storage time on the microorganisms count and physicochemical properties (pH, titratable acidity, syneresis and viscosity) of milk fermented with *L. casei* was carried out every two weeks during a storage period of 42 days. Throughout the period studied at 4 °C, no significant differences were found in terms of viscosity values, syneresis rates and *L. casei* counts, despite the substantial decrease in the pH values. Notably, the substantial increase in the concentration of free hydronium ions (active acidity) in the fiber-enriched matrices during the follow-up period was positively correlated with the *L.casei* survival. The microbial count determined in all samples was higher than 1 × 10^7^ CFU/g, the minimum value recommended by world organizations for nutraceutical fermented foods. Consequently, the studied prebiotic fibers could be considered in the production of new fermented dairy products with functional properties.

**Graphical abstract:**

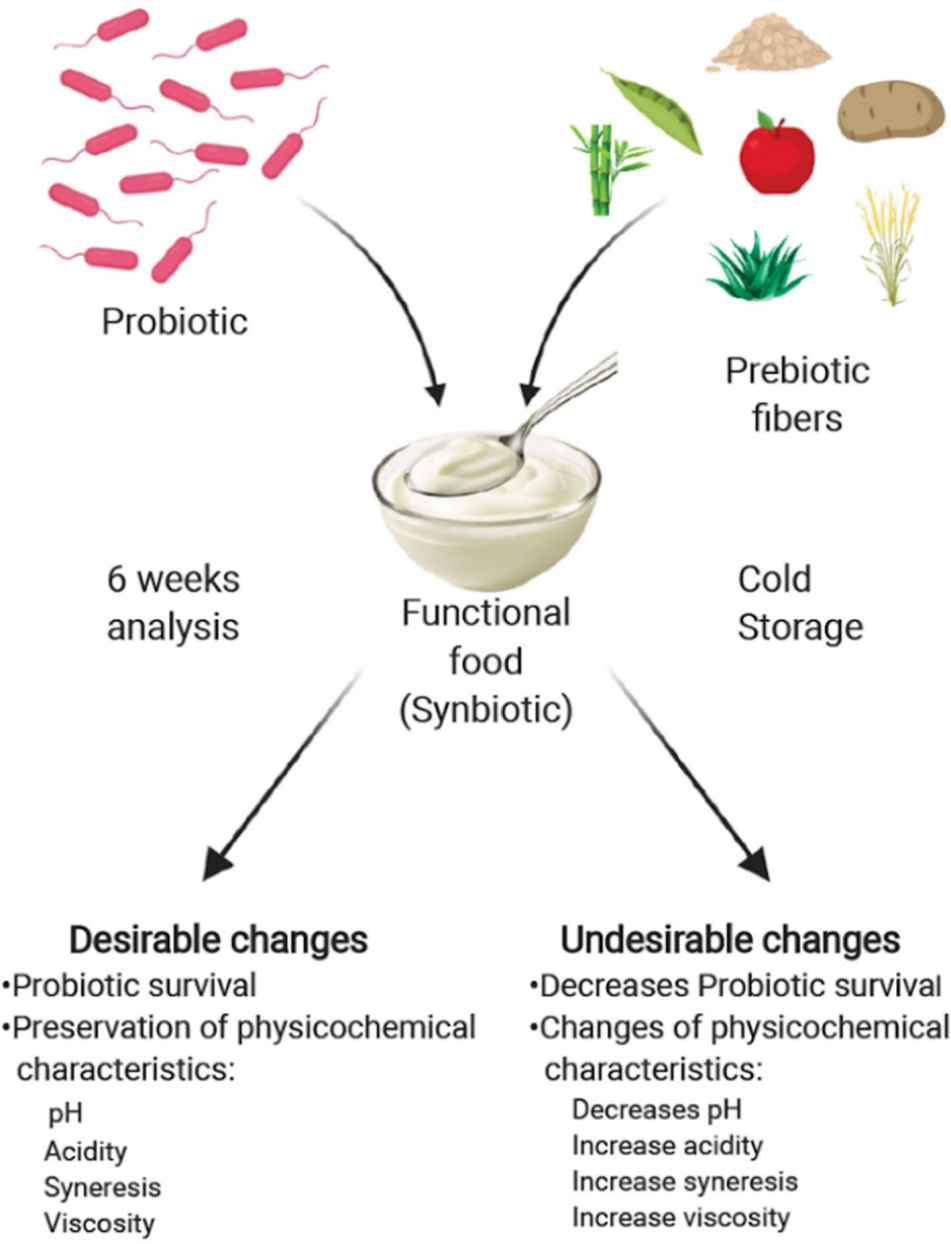

## Introduction

The probiotics have been defined as “live microorganisms, which when consumed in adequate amounts confer benefit to the host's health”, however recently, Zendeboodi et al. (2020), conceptualized a new definition of probiotics viable or unviable microbial cell (vegetative or spore; intact or ruptured) that is potentially healthful to the host. Among the probiotic bacteria found in the gastrointestinal microbiota, *L. casei* has been one of the best-studied species due to its commercial, industrial, and health wide potential (Amatayakul et al. 2019; AOAC 2006).

*L. casei* has wide applications in the production of fermented food products, different dairy products, and bioactive peptides in fermented milks, as a food additive (improving flavor and texture), as a starter culture, other fermented beverages; whey-based functional beverages, vegetable-based, just as cabbage, beet-and fruits; cantaloupe, cashew apple, and pineapple miscellaneous; quinoa and soy and also used in the pharmaceutical and cosmetic industry (García et al. 2019; Abdel-Hamid et al. 2019).

Notably, its beneficial bioactivity has been associated with various health-promoting functions, highlighting the regulation of intestinal microbiota (Sidira et al. 2010; Marinaki et al. 2016), improvement of innate immunity, reduction of pathogen-induced inflammation in addition to promoting different processes of intestinal homeostasis, namely the survival of intestinal epithelial cells, barrier functions and protective responses (Oelschlaeger 2010). Likewise, this strain has been found beneficial in decreasing cholesterol levels (Lye et al. 2010), reducing the propensity for obesity and diabetes, in addition to triggering pro-apoptotic and antiproliferative effects (Zhu et al. 2011; Grom et al. 2020), as well as reduced risk to suffer osteoporosis (Kim et al. 2009), and neurodegenerative diseases (Hill et al. 2018).

Since prebiotic fibers are a substrate selectively utilized by host microorganisms to confer health benefits, they become highly essential nutrients for healthy living. The non-digestible prebiotic fibers, such as polysaccharides, pectin, fructooligosaccharides, xylooligosaccharides, oligosaccharides, and inulin-type fructans (Mohanty et al. 2018; Mano et al. 2018; Colantonio et al. 2020) found in potato, apple, wheat, bamboo, oat, pea fiber, and carbohydrates, respectively.

However, despite the growing interest in nutraceutical foods, there are no conclusive results on the unique effect of fiber in the formulation of new products with functional properties. The differences found have been attributed to their unique physicochemical properties, which, when combined with probiotic microorganisms and food matrices of varied composition, affect the formulation's final properties that influence the probiotic microorganism's survival.

Therefore, herein we are interested in evaluating the physicochemical variables that could influence the survival of *L. casei* at 4 °C for a period of six weeks in fermented milk enriched with 3% non-digestible prebiotic exogenous fibers (oat, bamboo, pea, inulin, apple, potato, and wheat) intended to produce yogurt. It stands out that the final count of colonies of *L. casei* determined in all the test samples is higher than 1 × 10^7^ CFU/g, the minimum value recommended by world organizations for nutraceutical fermented foods. Thus, our exploratory outcomes do not support substantial deleterious effects of fibers composition on the survival of the microorganism *L. casei* in the fermented milk matrix.

## Material and methods

### Milk

Whole cow´s pasteurized milk (protein 3.5%, total sugars 4.5%, total fat: 3.4%, 2.5% saturated fat, and 5.0% unsaturated fat) was procured from commercial sources.

### Dietary fibers

Dietary fibers used in the study (inulin, oat, bamboo, pea, apple, potato, and wheat) are all commercial VITACEL^R^ brand.

### Starter culture

The *L. casei* CDBB-B-382 used as a probiotic strain was obtained from the National Collection of Microbial Strains and Cell Culture of CINVESTAV-IPN, México. Before being used as a starter culture, the lyophilized stock culture was reactivated twice in sterile MRS broth at 37 °C for 24 h then adjusted to tube number 5 of the McFarland nephelometer standard (1.5 × 10^9^ CFU/g) using sterile isotonic saline solution (0.9%) as diluent.

### Other ingredients

The chemicals (viz. phenolphthalein, sodium hydroxide, standard buffer solutions pH = 7.0, 4.0, and 9.0), culture media, sugar, and agar (stabilizer, CT20-RML) used were trademarked and procured from Sigma Aldrich, Becton Dickinson, Zulka and CYTECSA, respectively.

### Preparation of fermented milk

Control and fiber-load fermented milk were prepared in triplicates using different batches of pasteurized whole cow's milk and raw materials (n = 3). The fermented milk manufacturing process was elaborated following the procedure established in the Official Mexican Standard NOM-181-SCFI/SAGARPA-2018 and Codex Standard for Fermented Milks (Codex Stan 243-2003), -denomination physicochemical and microbiological specifications, commercial information, and test methods—(Marshall 1992).

To prepare the fermented milk the following ingredients were used: milk (1 liter), sugar (saccharose 4.0%), fibers (inulin, oat, bamboo, pea, apple, potato, and wheat, 3.0%), folic acid (0.02%), and 1.5% stabilizer (CT20-RML, CYTECSA). In parallel, a control was carried out with all the mentioned ingredients except the fiber.

The whole cow's milk was heated to a temperature of 45.0 ± 0.5 °C, sugar and fiber were added, and stirred with an Oster brand hand mixer until a homogeneous mixture was obtained. The heating was continued until reaching a temperature of 80 ± 0.5 °C; it was maintained at that temperature for 10 minutes (heat treatment). After that time, a thermal shock was induced by rapid cooling until reaching 37 °C. The mixture was inoculated with 2.0% of the starter culture of *L. casei*, which contained (1.5 × 10^9^ CFU/g) and was prepared as previously indicated, stirred until the total incorporation of the inoculum; the mixture was incubated at 37 °C until reaching a titratable acidity percentage of 0.75 ± 0.03%. The fermented milk was stored at 4 °C for 6 weeks. The analysis of cell viability and physicochemical variables was carried out at intervals of 0, 2, 4, and 6 weeks of storage in the refrigerator.

### Microbial analysis

#### Quantification of *L. casei*

A sample of 10 mL of each fermented milk was mixed with 90 mL of sterile isotonic saline solution (0.9%). Tenfold serial dilutions were prepared by adding 1 mL of each dilution to 9 mL of sterile isotonic saline solution. Then, 0.1 mL of each dilution was inoculated in MRS agar during the surface plating method, and after incubation at 37 °C under anaerobic conditions, for 72 h. Plates containing 30–300 colonies were selected and counted. Colonies were reported as total microbial count (CFU/g) (AOAC 2005; Karimi et al. 2012).

### Physicochemical analyses

#### Titratable acidity

Titratable acidity in terms of percentage of lactic acid was determined according to the AOAC for fermented milk (AOAC 2006) and Dimitrellou et al (2017). A sample of 9.0 g was taken in an Erlenmeyer flask and mixed homogenously by adding 20 mL distilled water (25 °C). After the addition of 0.25 mL phenolphthalein indicator (1.0 g/100 mL), the mixture was titrated against 0.1 N sodium hydroxide with continuous stirring until a persistent pink color appeared.

### pH

The pH of the fermented milk samples was measured at 0, 2, 4, and 6 weeks of storage at 4 °C after tempered 25 g of sample at 20 °C by using a calibrated digital pH-meter brand HANNA instruments model PH2. Five replications of each measurement were carried out for each formulation and storage time.

### Syneresis

Syneresis was determined using the method described by Amatayakul et al (2006). In this case, 10 g of control sample or fermented milk samples of 0, 2, 4, and 6 weeks of storage under refrigeration storage (4 °C) was weighed on a Denver Instrument brand APX-153 electronic analytical balance, and centrifuged in a Beckman Model L-70 ultracentrifuge and JA-14 rotor (Beckman Instruments, Palo Alto, CA) at 2500 rpm for 20 min at 4 °C. The syneresis was reported as the percentage of syneresis in the sample, and it was calculated as:

$$\% Syneresis = \frac{{weight\,whey}}{{sample\,weight}} \times 100$$

### Viscosity

The viscosity of samples storage under refrigeration (4 °C) at 0, 2, 4, and 6 weeks was determined using a viscometer DV-E, Brookfield-USA at 5 °C using spindles No. 6 and 7 at a shear rate of 60 rpm; and the results were reported in centipoise (cP). In case of the samples labeled as 0 week, the viscosity determination was measured after a storage time of 24 h in refrigeration.

### Statistical analysis

All data are presented as mean ± standard error of the mean (SEM) (n = 3). The difference between changes in cell viability (CFU/g), titratable acidity (% lactic acid), pH, syneresis (%), and viscosity (cP), elicited in different groups, and times were compared by two-way ANOVA with repeated measures for one factor (A: Time; B: Fiber), followed by a post hoc by the Bonferroni test. A P value < 0.05 was considered statistically significant.

## Results and discussion

### Microbial analysis

#### Quantification of *Lacticaseibacillus casei*

**Fig. 1:**
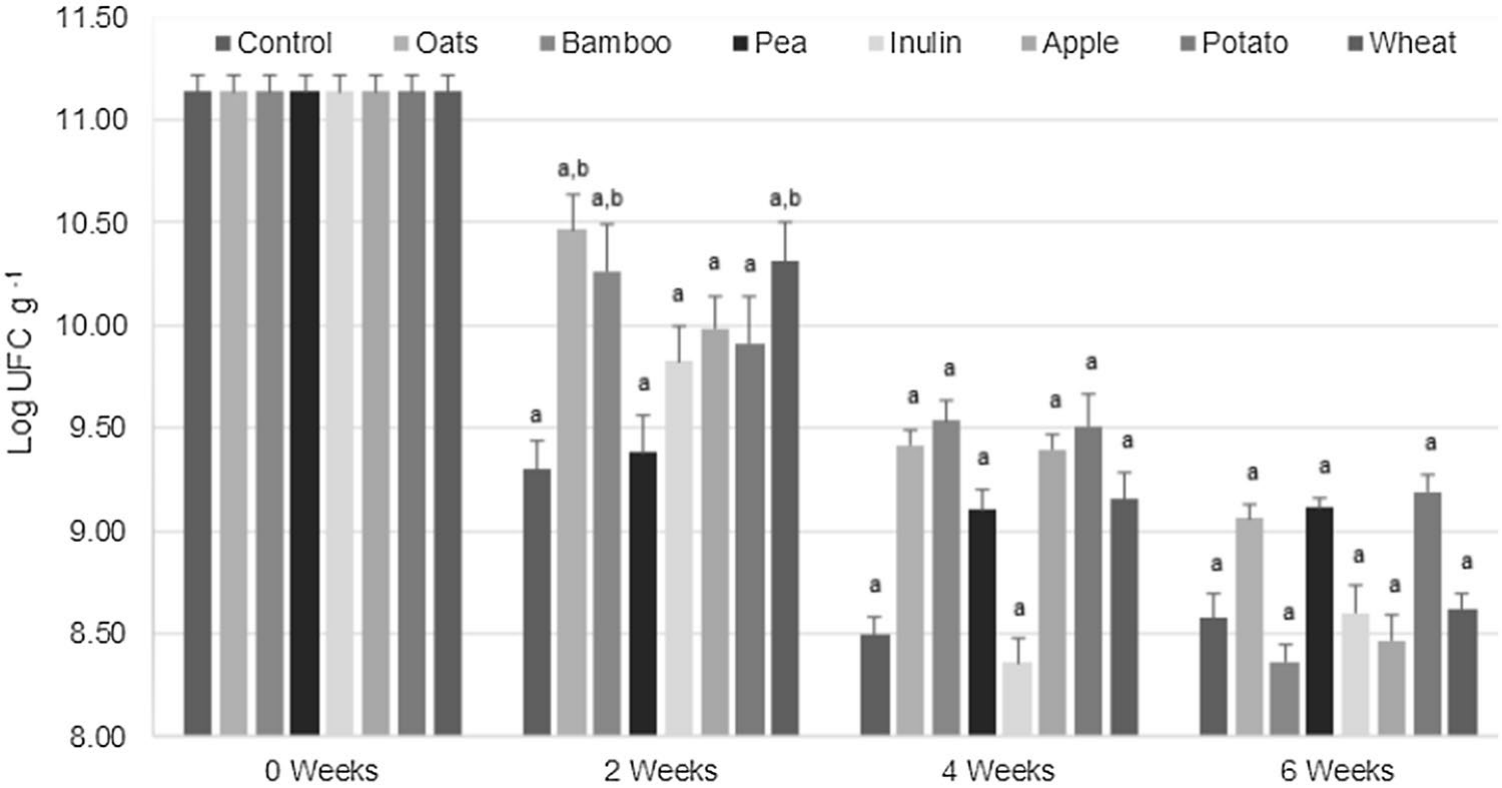
Results of viable counts of homofermentative *L. casei* colonies incorporated into the matrix of fermented milk enriched (3%) with non-digestible prebiotic fibers stored at 4 °C during six weeks period, **a** p < 0.05 versus week 0; **b** p < 0.05 versus control in the same week

The viable count of homofermentative *L. casei* used as starter culture (1.5 × 10^9^ CFU/g) in the fermented milk matrix containing 3% non-digestible prebiotic fibers (oats, bamboo, wheat, pea, inulin, apple, and potato) is shown in Figure 1.

Notably, the outcomes seem to depend largely on the susceptibility of the fibers to active bacterial metabolism determined by their specific chemical composition rather than on the prevailing pH condition (*vide infra*) in the matrix during the incubation period (Amatayakul et al. 2006; Savedboworn et al. 2017). Hence the rationalization for the statistically significant differences (p < 0.05) found in CFU/g values in 2 weeks between the control sample (9.30 ± 0.14 log CFU/g), oats (10.47 ± 0.17 log CFU/g), bamboo (10.27 ± 0.23 log CFU/g) and wheat fiber (10.31 ± 0.19 log CFU/g).

Notably, the outcomes at week six seem to be related to the high fiber content of cellulose and hemicellulose. For instance, bamboo fiber with a typical value of 74% cellulose and 26% hemicellulose induces a marked decrease in colony count (8.36 ± 0.09 log CFU/g) compared with pea fiber (9.11 ± 0.05 log CFU/g) containing a lower composition (62 %). Consequently, the inhibition of the growth of homofermentative *L. casei* could depend on the rate of degradation of these substrates to sugar that, after their evolution to lactic acid, alters the pH value of the matrix, modifying the ideal condition growth acidity (pH 6–7).

It should be noted that the CFU/g values determined in all samples are higher (1 × 10^8^ CFU/g) than the minimum value (1 × 10^7^ CFU/g) recommended by world organizations for nutraceutical fermented foods (FAO/WHO 2002; Codex Standard for Fermented Milks (Codex Stan 243-2003). Highlighting that oat, pea, and potato fibers exert a better influence on the survival capacity of the microorganism *L. casei* at 4 °C for a more extended period. This finding deserves special interest because oats are an essential ingredient of functional foods and an excellent source of β-glucan with preventive effects on various metabolic diseases. (Othman et al. 2011).

#### pH and titratable acidity

**Fig. 2:**
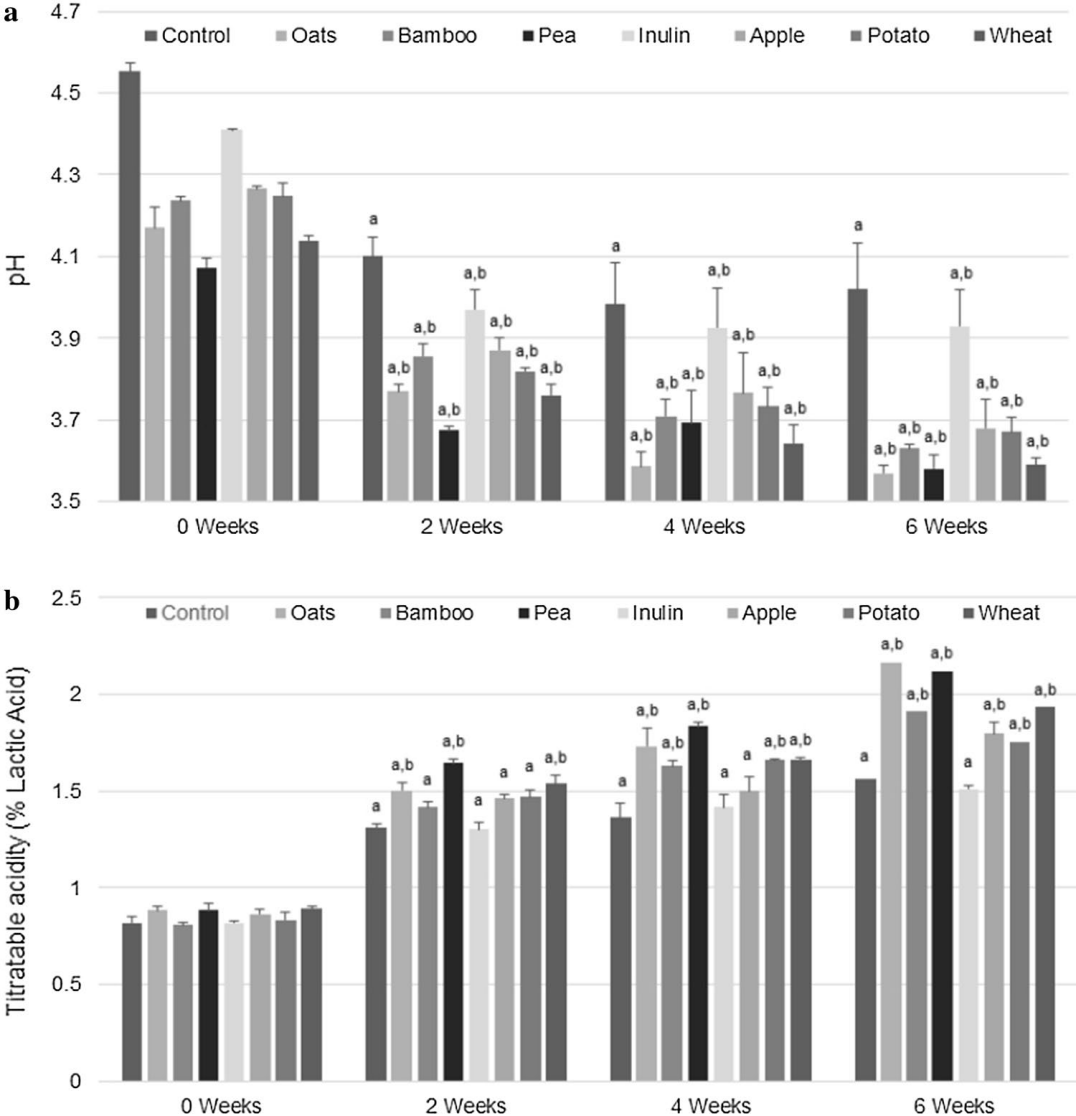
Mean values of pH (**a**) and titratable acidity (**b**, % lactic acid) for fermented milk enriched (3%) with non-digestible prebiotic fibers stored at 4 °C over a period of six weeks, **a** p < 0.05 versus week 0; **b** p < 0.05 versus control in the same week

The pH values and the lactic acid content are shown in Figures 2a, b. The pH of the products ranged between 3.59 and 4.55. The highest pH value was observed in the control sample, while the non-digestible fiber load decreased the pH values, evidencing a steady increase in acidity over time. The precise mechanism involved in this known effect is still elusive, but it could derive from production of acidic metabolites (such as short-chain fatty acids through the fermentation of undigested starch) (Naaeder et al. 1998; Liang et al. 2021) dependents of the soluble dietary fiber / insoluble dietary fiber ratio, combined with the intrinsic dietary fibers abilities to modify the microenvironment because of its water-holding capacity and protein chelating capacity (Poornima et al. 2020). All fiber-added formulations showed significant differences versus the control sample (p < 0.05) within the first two weeks of incubation. The most significant pH deviation was observed for the matrix added with peas, 3.68 *vs* 4.1. At the same time, the formulation based on inulin-type fructans, a mixture of polysaccharides and fructooligosaccharides, showed the highest pH value, comparable with the control experiment, 4.0 *vs* 4.1. Highlights that although the concentration of oxidanio ions changes over time, the variation in pH becomes less remarkable. Even though the oat-enriched matrix shows the lowest pH value (value comparable to pea and wheat at 6 weeks) after two weeks of incubation, the inulin-treated matrix exhibits the best ability to modulate hydronium ion (oxidanio) evolution over time; albeit with a slightly decreasing trend. Overall, oat, pea, and wheat fibers exert the minor ability to modulate oxidanio ions concentration over time (Figure 2a).

The titratable acidity of all the examined matrices containing 3% fiber differed and ranged between 0.81 ± 0.01 and 2.2 ± 0.01% lactic acid (Figure 2b). Thus, at two weeks, only the samples containing oat, pea, and wheat differed significantly from the control sample. The higher active and titratable acidity of fiber-added matrices compared to the control sample could be justified by the inherent fermentation activity of lactic acid bacteria during the incubation, depending on the intrinsic nature and composition of the fibers, of the fiber particle size, of the solubility and of the surface area exposed to bacterial degradation, as well as the post-acidification of products related to the continuity of the fermentative process. Thus, soluble dietary fibers, such as inulin, are digested by bacteria that generate acid metabolites, including lactic acid, which increase titratable acidity (Raju and Pal 2014). In addition to carbonic acids and hydronium ions that contribute to reducing the pH value (Mudgil 2017; Tyl and Sadler 2017). Thus, after two weeks of storage in the refrigerator at 4 °C fermented milks added with 3% fiber from oat, pea, and wheat fiber shows a statistical difference (p < 0.05) compared to bamboo, inulin, apple, and potato. As expected, the evolution of lactic acid increased over time in all cases, reaching its highest percentage of titratable acidity at the six weeks. The determination pH and lactic acid content ranged between 3.55 ± 0.09 and 4.05 ± 0.02 and between 1.5 ± 0.02 and 2.2 ± 0.01%, respectively. The results showed that only the inulin-based formulation did not show a statistically significant difference after two weeks of storage, which could mean a lower metabolic activity during the fermentation process because its molecular structure remained stable under such conditions during the cold storage period. This allegedly increased its ability to retain its structure unchanged (which is affected in the range pH 2.7−3.3 depending on the temperature) at a pH of 4.05 and a temperature of 4 °C over the shelf life of the product becomes a highly desirable feature because it can contribute to the structural stability of the matrix, and ultimately delay its degradation, influencing its final acceptability. In this sense, the decreasing percentage of titratable lactic acid determined during the follow-up time for all samples is as follows: oat (2.160 ± 0.104) ≈ pea (2.120 ± 0.035) > wheat (1.935 ± 0.016) ≈ bamboo (1.910 ± 0.018)> apple (1.795 ± 0.062) ≈ potato (1.760 ± 0.042) > control (1.560 ± 0.094) ≈ inulin (1.510 ±0.018).

#### Syneresis

**Fig. 3:**
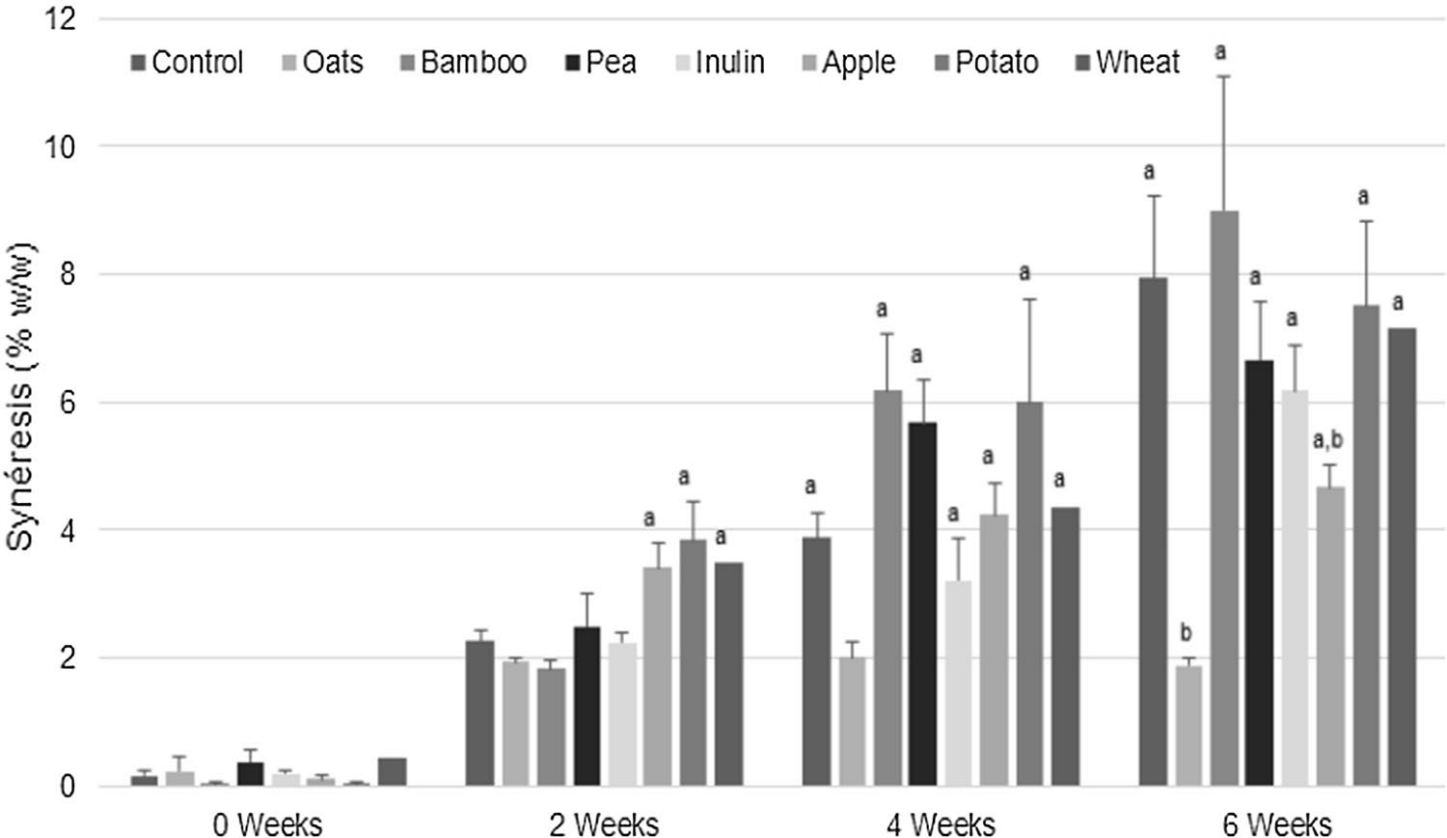
Syneresis expressed as percentage weight (% w/w) between control matrix and fermented milk added with 3% different fibers, **a** p < 0.05 versus week 0; **b** p < 0.05 versus control in the same week

Since syneresis is an undesirable characteristic in stored processed dairy foods, becoming one of the main visible defects that could lead to its rejection or acceptability (Amatayakul et al. 2006; Ghaderi‐Ghahfarokhi et al. 2020), the whey content was determined for the seven matrices of interest (Fig. 3). The percent of syneresis ranged between 0.033 ± 0.05 and 9.00 ± 1.80. It should be noted that among all the examined dietary foods, only oat and apple fibers showed statistically significant differences after a 6-weeks incubation period. However, the differences in the syneresis of the products cannot be attributed only to the increases in proton concentration induced by active bacterial metabolism over time but also to the intrinsic composition of the fiber itself. Accordingly, the oat-enriched fermented matrix releases the lowest volume of whey (1.883 ± 0.117%) throughout the follow-up period, while the bamboo-added matrix exhibits the highest syneresis effect (8.567 ± 0.333%); despite their slight difference shown in pH (~Δ 0.7). This means there are no significant differences in the syneresis values induced only by the pH conditions. This unfavorable effect could be attributed to its limited ability to immobilize water molecules within its microstructure due to its natural lipophilic intrinsic composition (lignocellulosic biomass). The presence of aliphatic and aromatic functional groups in high concentrations in the lignin macromolecule disrupt the hydrogen bonding formation (thus water absorption) while favoring Van der Waals interactions between the lipophilic components, thus generating a high syneresis effect. Therefore, the specific chemical structure of the fiber plays an essential role in mediating the physical properties, reducing the matrix's physisortive ability to create a looser gel with a lower water-holding capacity (Marinaki et al. 2016). This result indicated that the lower pH values might favor induced by the post-acidification during storage over time (from 4.25 ± 0.03 to 3.65 ± 0.03, vide supra) (Amatayakul et al. 2006; Ghaderi‐Ghahfarokhi et al. 2020).

#### Viscosity

**Fig. 4:**
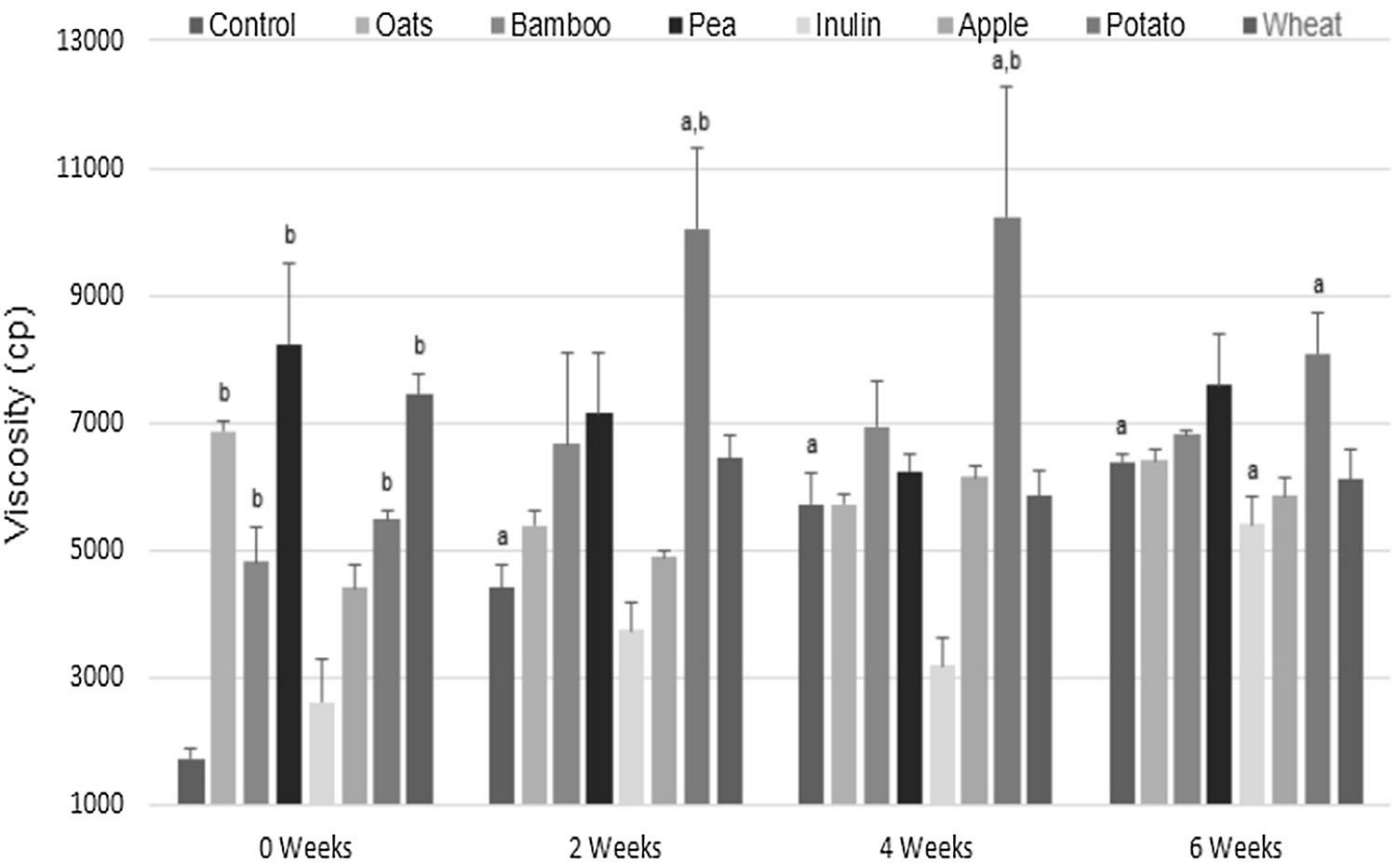
Determined viscosity (cP) for fermented milk added (3%) with non-digestible prebiotic fibers hold at 4 °C over a period of six weeks, **a** p < 0.05 *versus* week 0; **b** p < 0.05 *versus* control in the same week

Among the main factors known to affect the viscosity of dairy products are the intrinsic components of the matrix and the starter culture (Jaster et al. 2018). According to our results (Fig. 4), the viscosity of the samples evaluated varies widely between 1734.4 ± 173.17 cP and 10244.44 ± 201.18 cP. The viscosity of freshly fiber-added matrix (0 weeks) is composition-dependent, and their viscosity values ranged between 1734.4 ± 173.17 cP and 8233 ± 128.34 cP, with the highest value determined for the pea-added matrix. However, after 2 to 4 weeks, the results changed, and now, the matrix added with potato fiber reached its highest rheological value at 10244.4 ± 201.18 cP, and the sample with non-viscous inulin reached the lowest viscosity values that ranged between 3755.56 ± 428.86 cP and 3188.89± 441.81 cP. Nevertheless, after 6 weeks of incubation, the former formulation moderately decreases its viscosity value to around 8088.89 ± 641.28 cP. In this case, the rheological difference found might be attributed to variations in the intrinsic water retention capacity, which is a time-dependent physisortive phenomenon, and to the pH condition induced by the active fermentation (considering that its maximum water binding capacity reported at pH 5−7 is 15g H_2_O/g) (Lee et al. 2018). Hence, a higher swelling power improves the gelatinization effect that better limits water mobility at a low oxidanio ion concentration, leading to higher viscosity (Dikeman and Fahey 2006). It should be noted that the statistical results show that, unlike fermented milk with added oats for six weeks, no significant differences were found (p > 0.05) concerning the control sample. The order of viscosity increase (cP) determined after a period of six weeks is as follow: inulin (5411.1 ± 419.1) < apple (5866.7 ± 285.8) < wheat (6111.1 ± 472.7) < oats (6411.1 ± 164.5) < control (6400.0 ± 125.8) < bamboo (6811.1 ± 84.1) < pea (7600.0 ± 784.6) < potato (8088.9 ± 641.3). Although several factors influence the final viscosity of dietary fibers in solution, the viscosity order found suggest that the dietary fibers that have a high percentage of soluble fiber correlate positively with a higher viscosity degree as denoted by the value determined by the matrix containing potato fiber (10%) *vs *the wheat fiber (2.5%). It is also inferred that the improvement in the viscosity of the inulin-loaded matrix over time (0-week *vs* 6 weeks) may be due to its gelling ability due to its supramolecular chemistry (Barclay 2010).

## Conclusions

To improve the health benefits of dairy products, seven available commercial brand dietary fibers were incorporated into fermented milk and the effects of physicochemical parameters overtime on the survival of the microorganism *L. casei* was assessed. Significant differences were found in all the samples evaluated in terms of active and total acidity, except in the values of viscosity, syneresis percentage, and count of microorganisms. In particular, the increase in oxidanio ions concentration over time was positively correlated with *L. casei* growth at a specific fiber load, as indicated by the total colony count found to be greater than 1x10^7^ CFU/g. Due to their beneficial influence on the survival of *L. casei*, these fibers could be considered essential ingredients that could incorporate healthy fibers into the human diet through functional foods. Therefore, they could promote the technological development of new fermented milk products with functional properties capable of preventing profound alteration in the intestinal microflora. Therefore, this future field of research could range from the food industry to the pharmaceutical industry, particularly in the field of disorders related to the gastrointestinal tract.

## Data Availability

Raw data were generated at the [Facultad de Estudios Superiores Cuautitlán, UNAM.] large-scale facility. Derived data supporting the findings of this study are available from the corresponding author upon request.
